# Antiphospholipid Antibodies and Systemic Scleroderma

**DOI:** 10.4274/Tjh.2013.0127

**Published:** 2013-12-05

**Authors:** Muhammed Mubarak, Hamid Nasri

**Affiliations:** 1 Sindh Institute of Urology and Transplantation (SIUT), Karachi, Pakistan; 2 Isfahan University of Medical Sciences, Department of Nephrology, Division of Nephropathology, Isfahan, Iran

## DEAR EDITOR

We read with great interest the article by Touré and colleagues published in a recent issue of your journal, entitled “Antiphospholipid Antibodies and Systemic Scleroderma”. In this study, they investigated 40 patients with systemic scleroderma (SSc) for the presence of antiphospholipid antibodies (aPLs). Overall, they found aPLs in 23 (57.5%) patients. The most frequently encountered antibody was IgG anti-β2 glycoprotein I (GP I) (37.5% of the patients), followed by anticardiolipins (17.5%) and lupus anticoagulants (5%). No statistically significant association of positive aPL tests to any of the scleroderma complications was observed. They suggested that patients affected by SSc be followed in order to monitor vascular complications following the confirmation of the presence of antiphospholipid syndrome (APS) [1]. We would like to remind readers of a few points about APS nephropathy (APSN) and rheumatologic disease. Although high proportions of patients showing association of SSc and aPLs were described by Touré et al., more attention and support is needed to find the co-existence of APSN with systemic lupus erythematosus and SSc in routine evaluation of these patients [2,3,4]. Thus, the main question to bear in mind is: what do nephrologists or rheumatologists need to know about APS and APSN? 

APS is being increasingly recognized as an important cause of renal damage due to thrombosis at any location within the renal vasculature [5]. The term “APSN” refers to the various renal pathologies caused by vascular lesions in the glomeruli, arterioles, and/or interlobular arteries in patients with aPLs [5,6,7]. From the nephrology point of view, this small-vessel, vaso-occlusive nephropathy may present with hypertension, acute and/or chronic renal failure, and often a low-grade proteinuria clinically [8,9,10]. It is also necessary to increase the awareness of the morphologic features of this disease among pathologists and nephrologists from developing countries for its early and accurate diagnosis and appropriate management. It is clear that the diagnosis of APSN can only be made based on renal biopsy. None of the patients in the study under discussion showed renal manifestations or complications, and neither did they undergo renal biopsy [11,12]. 

There were additionally some errors in the study. The authors did not classify the disease. The timing of antibody testing was also not provided. It is not clear how many of the patients were diagnosed with APS. Laboratory criteria alone are not enough to definitively diagnose this condition. The titer of antibodies was not given. This may be one of the factors underlying the lack of association of the aPLs with complications of SSc. There was also no information about SSc serologies such as anti-Sc70. An age of 41 years is given as both the median and mean value in different places. The frequency of anti-β2 GP I is given as 50% in the results section, but it is 37.5% as given in Table 2. Hypochromic skin macula is described as present in 100% of cases in the results section, while in Table 1, it is 94.7%. The percentage value of pulmonary hypertension in Table 1 is also wrong; it should be 12.5%. The values for complications/manifestations in Tables 1 and 3 do not match. In the discussion section, the study period is given as 8 months rather than 18 months. 

## DECLARATION OF CONFLICTING INTERESTS

The authors declare no conflicts of interest with respect to the authorship and/or publication of this article.
